# A Systematic Review and Methodological Evaluation of Published Cost-Effectiveness Analyses of Aromatase Inhibitors versus Tamoxifen in Early Stage Breast Cancer

**DOI:** 10.1371/journal.pone.0062614

**Published:** 2013-05-06

**Authors:** Ava A. John-Baptiste, Wei Wu, Paula Rochon, Geoffrey M. Anderson, Chaim M. Bell

**Affiliations:** 1 Women’s College Research Institute, Women’s College Hospital, Toronto, Ontario, Canada; 2 Keenan Research Centre in the Li Ka Shing Knowledge Institute, St. Michael’s Hospital, Toronto, Ontario, Canada; 3 Pharmacoeconomics Research Unit, Cancer Care Ontario, Toronto, Ontario, Canada; 4 Canadian Centre for Applied Research in Cancer Control, Toronto, Ontario, Canada; 5 Institute for Clinical Evaluative Sciences, Toronto, Ontario, Canada; 6 Institute of Health Policy, Management and Evaluation, Faculty of Medicine, University of Toronto, Toronto, Ontario, Canada; 7 Department of Medicine, Mount Sinai Hospital, Toronto, Ontario, Canada; Dartmouth, United States of America

## Abstract

**Background:**

A key priority in developing policies for providing affordable cancer care is measuring the value for money of new therapies using cost-effectiveness analyses (CEAs). For CEA to be useful it should focus on relevant outcomes and include thorough investigation of uncertainty. Randomized controlled trials (RCTs) of five years of aromatase inhibitors (AI) versus five years of tamoxifen in the treatment of post-menopausal women with early stage breast cancer, show benefit of AI in terms of disease free survival (DFS) but not overall survival (OS) and indicate higher risk of fracture with AI. Policy-relevant CEA of AI versus tamoxifen should focus on OS and include analysis of uncertainty over key assumptions.

**Methods:**

We conducted a systematic review of published CEAs comparing an AI to tamoxifen. We searched Ovid MEDLINE, EMBASE, PsychINFO, and the Cochrane Database of Systematic Reviews without language restrictions. We selected CEAs with outcomes expressed as cost per life year or cost per quality adjusted life year (QALY). We assessed quality using the Neumann checklist. Using structured forms two abstractors collected descriptive information, sources of data, baseline assumptions on effectiveness and adverse events, and recorded approaches to assessing parameter uncertainty, methodological uncertainty, and structural uncertainty.

**Results:**

We identified 1,622 citations and 18 studies met inclusion criteria. All CE estimates assumed a survival benefit for aromatase inhibitors. Twelve studies performed sensitivity analysis on the risk of adverse events and 7 assumed no additional mortality risk with any adverse event. Sub-group analysis was limited; 6 studies examined older women, 2 examined women with low recurrence risk, and 1 examined women with multiple comorbidities.

**Conclusion:**

Published CEAs comparing AIs to tamoxifen assumed an OS benefit though none has been shown in RCTs, leading to an overestimate of the cost-effectiveness of AIs. Results of these CEA analyses may be suboptimal for guiding policy.

## Introduction

There is growing concern over the ability, even in high income countries, to deliver affordable cancer care. [1], [2] Cost-effectiveness analysis (CEA) is recognized as an important tool for assessing value for money and an important source of information for making clinical and policy decisions. CEA can play a central role in guiding appropriate resource allocation in cancer care. [1], [2].

Cost-effectiveness analysis (CEA) is the comparative assessment of two or more interventions in terms of costs and benefits. [3] CEA is frequently used by organizations responsible for financing health care to quantify the value for money associated with adopting a new therapy compared to continued use of an existing therapy. The main output of a CEA is the incremental cost-effectiveness ratio or ICER. The ICER is the ratio of increased health expenditures divided by increased health outcomes when a new therapy is compared to an existing therapy. Health outcomes can be measured in terms of life expectancy and the resulting ICER, represents the ratio of increased expenditures to increased years of life. There is no consensus on the threshold ICER value, above which a new therapy is considered too expensive although the threshold of $100 K per life year has been commonly cited. [4] In reality, decision makers in different jurisdictions employ different criteria as threshold values. [5] A preferred measure of health outcomes, the quality-adjusted life year (QALY), is derived by weighting life expectancy on a scale ranging from 0 to 1, known as a utility. A utility indicates the desirability of a health state based on morbidity and quality of life impact. [6], [7] For example, living ten years with a chronic disease that has a utility of 0.7 is the equivalent of living 7 quality adjusted life years (QALYs). CEAs in which the outcome is measured in terms of QALYs produce an Incremental Cost Utility Ratio (ICUR), which incorporates quality of life impact into the estimate of value for money. The incremental cost per life year and incremental cost-utility ratios are collectively referred to as ICERs although this simplification can result in some confusion. Health state utilities can be estimated using a variety of methods, and different methods can result in different utilities and thus different QALY estimates. [6], [7].

In trial-based CEAs, information on health care costs and health outcomes are collected during the course of a randomized controlled trial (RCT). [3] The ICER is estimated based on the cost and health outcomes measured during the trial. More commonly, CEA estimates are derived from decision models. In model-based CEA, information on health care resource utilization, costs and health outcomes are derived from a variety of potential sources including RCTs, meta-analyses, observational studies, medical record review, patient interviews and the opinions of expert panels. The information is incorporated into a mathematical model. [8] Model-based CEAs typically extrapolate short term data available from RCTs or observational studies into longer term outcomes to estimate an ICER or ICUR. Model-based CEA provides the opportunity to clearly identify key assumptions and vary model components to understand the impact of uncertainty on estimates of value for money. Sensitivity analysis is the systematic variation in a model input to assess the impact on model outputs and it is crucial for ensuring the validity of model-based CEA estimates. [9].

Cancer drug therapies are well studied in randomized controlled trails (RCTs) and RCTs are a key source of evidence incorporated into cancer CEAs. However, there are limitations to the data from RCTs. One issue is that they often use surrogate endpoints rather than overall survival (OS). Improvements in survival are a clear benefit to patients whereas the benefits of surrogate endpoints such as DFS are less clear. The studies evaluating five years of aromatase inhibitors compared to five years of tamoxifen in the treatment of post-menopausal women diagnosed with early stage hormone receptor positive breast cancer used disease free survival (DFS) rather than OS. Aromatase inhibitors reduced the risk of breast cancer recurrence when compared to tamoxifen. [10], [11] However, follow-up data reveal that the increased DFS associated with AIs in this setting does not translate into increased OS. [12], [13].

Another well recognized problem is that RCTs of cancer drugs often do not provide detailed or complete data on adverse effects. [14] Tamoxifen and aromatase inhibitors have different side effect profiles. Tamoxifen is associated with an increased risk of endometrial cancer and thromboembolism and AIs are associated with an increased risk of fractures. [15], [16] The lack of survival benefit for women treated with AIs compared to tamoxifen has raised concerns that AI-related adverse events may mitigate the benefits of reduced breast cancer recurrence. [17], [18] Recent evidence supports the idea that the relative impact of adverse events between AIs and tamoxifen should be investigated further. A systematic review and meta-analysis of RCTs demonstrated that while AIs are associated with lower risks of venous thromboembolism and endometrial cancer, they are also associated with increased risks of fractures and cardiovascular disease when compared to tamoxifen. [12] Finally, there are issues of generalizability. Individuals in the RCT may be different than individuals treated in the real-world who may be older or have more co-morbidity, factors with important impacts on risks and benefits. Particular sub-groups, such as older women, those at low risk of breast cancer recurrence, or those at high risk of fracture may be especially vulnerable. As a result, the added benefit of AI therapy when compared to tamoxifen in real-world practice has been called into question. [18].

This paper provides a systematic review of CEA studies of AI versus tamoxifen. We describe the overall quality of these studies, focusing specifically on how uncertainty is assessed and its potential impact on study conclusions and policy implications.

## Materials and Methods

### Systematic Search and Identification of Relevant Studies

We conducted a systematic review of the published literature to identify CEAs addressing first line hormonal therapy for early stage breast cancer in women. We searched the following electronic databases: MEDLINE (1996–March 9 2011), EMBASE (1996–February 2011), PsychINFO (1996–March Week 1 2011), and the Cochrane Database of Evidence-Based Medicine Reviews (1996–February 2011). (Appendix S1) We set the lower limit of our literature search to 1996 prior to the beginning of RCTs assessing aromatase inhibitors in early stage breast cancer. We included CEAs addressing the patient population of post-menopausal women diagnosed with early stage breast cancer, comparing at least one aromatase inhibitor to tamoxifen, considering first line hormonal therapy, and expressing health outcomes in terms of life years or quality adjusted life years (QALYs). No language restrictions were employed. Non-English articles were translated using Google Statistical Machine Translation. [19] We excluded CEAs addressing planned or unplanned switching from tamoxifen to aromatase inhibitor, or extended adjuvant therapy. We excluded descriptive costing studies with no consideration of health outcomes as these are not considered full CEAs.

Two reviewers (AJB, WW) independently screened titles and abstracts of identified citations for relevance. Full texts of potentially relevant articles were retrieved and independently screened by both reviewers. Disagreements were resolved through consultation with a third reviewer (CB).

### Quality Appraisal

We appraised the studies according to general guidelines for the conduct and reporting of cost-effectiveness analysis using the tool developed by Neumann et al. [20] The instrument assesses quality in five different domains: framing, reporting of costs, reporting of results, discussion and overall assessment. Items in the framing domain include disclosure of the funding source, statement of study perspective, listing of modeling assumptions, inclusion of a model diagram and reporting of the discount rate. Items under reporting of cost items include reportion of net costs, source of valuation, year of monetary units, preference weights, preference measurement technique and source of preferences. Items under the reporting of results domain include appropriate reporting of incremental analyses, sensitivity analysis of costs, preference weights, estimates of effectiveness and discount rate. Discussion items include discussion of study limitations, ethical implications and comparison of results to other anaylyses. The overall subjective assessment of quality is rated out of 7. We averaged the subjective quality rating between two reviewers.

### Data Abstraction

We abstracted characteristics of the identified studies including publication year, country, comparators, outcomes (life years, QALYs, or both), model perspective, type of model, study sponsorship, time horizon and discount rate.

### Model Outputs

We abstracted incremental cost effectiveness ratios (ICERs) and the incremental cost-utility ratio (ICUR) from each study. To allow direct comparison across countries and years we converted the ICERs and ICURs to a common year and currency (2010 US Dollars). We first converted to US dollars using the Purchasing Power Parities (PPPs) for health from the World Bank. [21], [22] This approach has been employed in other studies comparing CEAs across countries. [23], [24] We then converted to 2010 US Dollars using the U.S. Consumer Price Index. [25] We also abstracted the survival benefit the models estimated for aromatase inhibitors compared to tamoxifen.

### Approach to Addressing Uncertainty

We used a previously identified framework to characterize uncertainty in the context of CEA. Uncertainty can be divided into three categories : 1) parameter uncertainty, 2) structural uncertainty, and 3) methodological uncertainty. [26] Parameter uncertainty is uncertainty about the true numerical values of input parameters. [26] For example, there may be uncertainty about the estimate of the impact of AIs on the risk of breast cancer recurrence. Structural uncertainty is uncertainty about the correct way to combine the parameters of the model. [26] For example, uncertainty in modeling adverse events is an example of structural uncertainty, including which events to model, and what downstream effects of adverse events to incorporate. Methodological uncertainty refers to choices about population, time horizon, and study perspective that impact how CEA estimates are calculated. [26], [27] For example, the method for extrapolating short-term data from the trial on rates of recurrence to longer-term estimates is an example of methodological uncertainty.

### Data Sources and Parameter Uncertainty

We abstracted the source of data on breast cancer recurrence risk and harms associated with hormonal therapies (single RCT, meta-analysis, risk model, observational data, or a combination of sources). For example, relative risk estimates from RCTs could be combined with observational data on baseline risk that better reflects patients in practice. [28], [29] We critiqued the authors’ handling of parameter uncertainty by determining whether authors performed sensitivity analyses on parameters specifying the relative risk of breast cancer recurrence and the relative risk of adverse events, including fractures, cardiovascular events, stroke, thromboembolism, and endometrial cancer. We also critiqued the authors’ handling of parameter uncertainty by determining whether the authors performed probabilistic sensitivity analysis or conducted value of information analysis to determine if there was enough uncertainty in the analyses to warrant investigating AIs through additional research. [30].

### Structural Uncertainty

We assessed the authors handling of structural uncertainty by abstracting what adverse events the authors incorporated into the CEA models and recording whether or not increased mortality following adverse events was incorporated into the models. For example, a systematic review of the literature demonstrated that for older patients mortality rates can increase more than five-fold in the three months following hip fracture and an elevated risk of death lasts for many years. [31].

### Methodological Uncertainty

We assessed methodological uncertainty by determining whether or not authors conducted scenario analyses or sub-group analyses to look at cost-effectiveness in special populations, such as older women, women with co-morbidities or women at high risk of fracture. We also assessed methodological uncertainty by determining whether or not the authors performed sensitivity analysis on the discount rate or the method of extrapolating short term trial data over the long-term.

Two reviewers (AJB, WW) performed data abstraction and quality appraisal, with disagreements resolved through consultation with a third reviewer (CB).

## Results

### Literature Search

**Figure 1:**
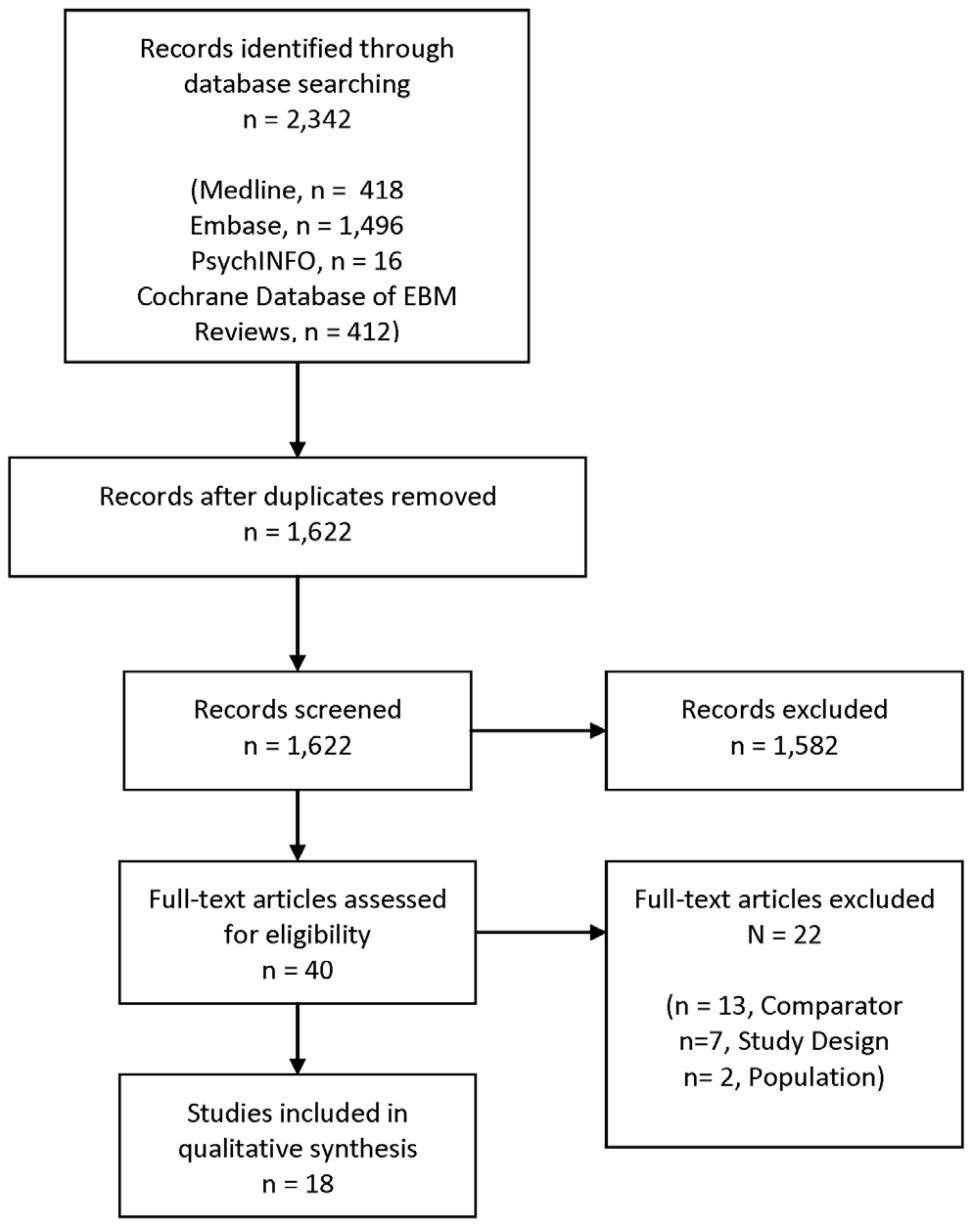
PRISMA Flow Diagram. PRISMA indicates Preferred Reporting Items for Systematic Reviews and Meta-Analyses.

We identified 1,622 non-duplicate citations, of which 40 were recognized as potentially relevant and the full text articles retrieved. (Figure 1) Of the total of 40 studies, 13 were excluded because of the comparators, seven were excluded because the study was not a cost-effectiveness analysis, and two were excluded because the study population was not early breast cancer.

### Study Characteristics

A total of 18 articles were included in the final study, of which 16 were published in English, one in Spanish and one in Italian.[32]–[49] The publication years ranged from 2004 to 2010. Most studies (5/18) addressed policy decision making in European zone countries, three each addressed the United States, United Kingdom, and Canada. (Table 1) Eleven of the studies acknowledged funding from industry and the funding source was not stated for three studies. Most analyses compared anastrazole to tamoxifen (n = 13) and the remainder compared letrozole to tamoxifen. All of the studies were model based – we identified no trial-based analyses. The vast majority of studies used Markov Cohort models (n = 17). In the remaining study by Lazzaro et al, the authors indicated that a model was used but the model type was unclear. Most considered both QALY and life year outcomes (n = 11). The majority of CEAs (n = 16) conducted the analyses from the perspective of the health care system as payer, accounting for the costs attributable to provider organizations such as government payers and excluding patient costs. One-half of studies used a 3% discount rate. Detailed information on study characteristics is available on-line in Table S1.

**Table 1:** Summary of study characteristics (N = 18).

Study Characteristics	
Country/Region	
Euro zone	5 (28%)
United States	3 (17%)
United Kingdom	3 (17%)
Canada	3 (17%)
Brazil	2 (11%)
Colombia	1 (6%)
Korea	1 (6%)
Publication Year	
2004–2007	11 (61%)
2008–2010	7 (39%)
Comparators	
Tamoxifen, Anastrazole	13 (72%)
Tamoxifen, Anastrazole,Letrozole	3 (17%)
Tamoxifen, Letrozole	2 (11%)
Perspective	
Health Care Payer	16 (89%)
Societal	1 (6%)
Multiple perspectives	1 (6%)
Type of Model	
Markov cohort model	17 (94%)
Unclear	1(6%)
Sponsorship	
Industry	11 (61%)
Government fundingagency	3 (17%)
Not Stated	3 (17%)
Other	1 (6%)
Outcomes	
QALYs and Life Years	10 (56%)
QALYs	6 (33%)
Life years	2 (11%)
Time Horizon	
Lifetime	2 (11%)
50 Years	1 (6%)
35 Years	2 (11%)
30 Years	3 (17%)
25 Years	4 (22%)
20 Years	5 (28%)
Less than 10 Years	1 (6%)
Discount Rate	
3%	9 (50%)
3.5%	3 (17%)
5%	3 (5%)
Other*	2 (11%)
Not Reported	1 (6%)

QALYs indicates quality adjusted life years.

* The discount rates in these studies was different for costs and benefits.

Detailed information on study characteristics is available in Appendix S2.

### Quality Appraisal

Detailed items on the Neumann critical appraisal instrument are available online in Table S2. Eighty-three percent of studies reported on the funding source. All CEAs clearly stated the study perspective and 17 of 18 (94%), listed modeling assumptions. The vast majority of study authors provided information on the source of cost estimates 17 of 18 (94%), but authors frequently overlooked reporting methods for measuring utilities. Among 16 studies estimating an ICUR, only seven (44%) provided information on the method for measuring utilities. Furthermore, only 7 of 16 studies (44%) performed sensitivity analysis on preference weights. The average subjective quality score on a scale of 1 to 7 was 3·5, ranging from 1 to 5·3.

### Cost-effectiveness Analysis Outputs

When comparing anastrazole to tamoxifen, ICERs ranged from $77 to $97,202 per life year and ICURs ranged from $7,351 to $151,608 per QALY. (Table 2) For the comparison of letrozole to tamoxifen, ICERs ranged from $24,109 to $61,278 per life year and ICURs from $25,886 to $59,620 per QALY. All ICERs from the published analyses were less than $100 K (2010 USD) per life years and the majority less than $50 K (2010 USD per life year. All ICURs with the exception of the analysis by Gil et al comparing anastrazole to tamoxifen were under the threshold of $100 K (2010 USD) per QALY and the majority under $50 K per QALY. All studies estimated a survival benefit for aromatase inhibitors compared to tamoxifen. For those studies reporting undiscounted life years gained, the mean (standard deviation) estimated increase in survival was 0·39 (0·22) years for anastrazole when compared to tamoxifen and 0·38 (0·30) years for letrozole compared to tamoxifen.

**Table 2:** Cost-effectiveness analysis outputs.

No.	Author	Undiscounted life years gained		ANA vs TAM		LET vs TAM	
		(ANAvs TAM)	(LET vs TAM)	ICER	ICUR	ICER	ICUR
1	Delea^1^	0·68		−	−	$22,209 USD 2005 $24,797 USD 2010	$23,743 USD 2005 $26,509 USD 2010
2	Delea^2^	0·77		−	−	$22,038 CAD 2005 $24,109 USD 2010	$23,662 CAD 2005 $25,886 USD 2010
3	Fonseca^3^			R$27,327 BRL 2005 $42,870 USD 2010	−	−	−
4	Gamboa^4^	0·49		$37,071 COP 2007 $77 USD 2010	−	−	−
5	Gil^5^	0·54		€33,282 EUR 2004 $80,763 USD 2010	€62,477 EUR 2004 $151,608 USD 2010	−	−
6	Hillner^6^	0·17		$40,600 USD 2002^a^ $49,211 USD 2010	$75,900 USD 2002^a^ $91,998 USD 2010	−	−
7	Hind^7^	0·08	0·16	£36,225 GBP 2004 $97,202 USD 2010	£31,965 GBP 2004 $85,771 USD 2010	£22,837 GBP 2004 $61,278 USD 2010	£21,580 GBP 2004 $57,905 USD 2010
8	Karnon^8^	0·42	0·59	£11,703 GBP 2005 $30,373 USD 2010	£11,428 GBP 2005 $29,660 USD 2010	£10,502 GBP 2005 $27,256 USD 2010	£10,379 GBP 2005 $26,937 USD 2010
9	Lazzaro^9^			−	€47,556 EUR 2005 $74,868 USD 2010	−	−
10	Lee^10^			−	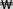 22,461,689 KRW 2009 $63,757 USD 2010	−	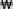 21,004,142 KRW 2009 $59,620 USD 2010
11	Locker^11^	0·22		$23,541 USD 2003 $27,898 USD 2010	$20,246 USD 2003 $23,993 USD 2010	−	−
12	Lux^12^				€21,069 EUR 2008 $37,181 USD 2010	−	−
13	Mansel^13^	0·23		£18,702 GBP 2004 $50,183 USD 2010	£17,656 GBP 2004 $47,376 USD 2010	−	−
14	Moeremans^14^	0·35		€4,233 EUR 2004^a^ $7,862USD 2010	€3,958 EUR 2004^a^ $7,351 USD 2010	−	−
15	Rocchi^15^	0·39		$30,000 CAD 2004 $33,931 USD 2010	$28,000 CAD 2004 $31,669 USD 2010	−	−
16	Sasse^16^				R$ 32,403 BRL 2005 $50,834 USD 2010	−	−
17	Skedgel^17^				$27,622 CAD 2005 $30,218 USD 2010	−	−
18	Skedgel^18^				€19,982 EUR 2005 $35,897 USD 2010	−	−

### Data Sources and Handling of Parameter Uncertainty

The majority of CEA authors took estimates of the impact of hormonal therapies on breast cancer recurrence from a single RCT, without developing a natural history model based on other data sources. (n = 14) (Table 3) Data were taken directly from either the Arimidex or Tamoxifen Alone or in Combination (ATAC) trial which compared five years of anastrazole to five years of tamoxifen or the Breast International Group (BIG 1–98) trial which compared five years of letrozole to five years of tamoxifen. A total of 10 studies (56%) reported sensitivity analysis on the risk of breast cancer recurrences, meaning a significant proportion did not report sensitivity analyses on this important factor.

**Table 3:** Summary of data sources and handling of uncertainty (N = 18).

Category	
Data sources	
Recurrence rates	
Single RCT	14 (78%)
Combination of data sources^a^	4 (22%)
Meta-analysis	0 (0%)
Adverse event rates	
Single RCT	9 (50%)
Meta-analysis	0 (0%)
Combination of data sources	8 (44%)
Other	1 (6%)
Handling of parameter uncertainty	
Performed sensitivity analysis on the risk of breast cancer recurrence	10 (56%)
Performed sensitivity analysis on the adverse events	12 (67%)
Performed probabilistic sensitivity analysis	11 (61%)
Performed value of information analysis	0 (0%)
Handling of structural uncertainty	
Incorporated increased mortality following any adverse event?	11 (61%)
Incorporated increased mortality following	
Fracture	6 (33%)
Cardiovascular Events	3 (17%)
Stroke	0 (0%)
Thromboembolism	4 (22%)
Endometrial Cancer	4 (22%)
Handling of methodological uncertainty	
Addressed the following sub-groups	
Older cohorts of women	6 (33%)
Women at low risk of breast cancer recurrence	2 (11%)
Women at high risk of fracture	1 (6%)
Women with high risk of cardiovascular disease	0 (0%)
Women at high risk of stroke	0 (0%)
Women at high risk of thromboembolism	0 (0%)
Women at high risk of endometrial cancer	0 (0%)
Women with multiple co-morbid diseases	1 (6%)
Performed sensitivity analysis on the method of extrapolating breast cancer recurrence rates beyond thefollow-up time of available studies (n = 17)^b^	11 (65%)
Performed sensitivity analysis on the discount rate	13 (72%)

a Authors combined observational data or a risk model with RCT data.

b One study did not extrapolate beyond the time horizon of the trial data used in construction of the model. (Lazzaro et al [38]).

Half of the studies took data on adverse events from a single RCT with the other half incorporating external information. (Table 3) For example, Hillner et al applied the increased hip fracture risk from the ATAC trial to age-specific data on hip fracture rates from Scandinavia. [37] In one study the authors cited multiple data sources for information on harms but it was unclear how the information was incorporated into the model. [40] One-third of the studies did not perform sensitivity analysis on the risk of adverse events (n = 6, 33%). Eleven studies (61% ) performed probabilistic sensitivity analyses. No studies performed a value of information analysis to quantify the value of more research to better estimate model parameters and better inform the policy decision. Detailed information on data sources and handling of parameter uncertainty is available in Table S3.

### Handling of Structural Uncertainty

A significant proportion of analyses (n = 7, 39%) assumed no additional mortality following any adverse event. (Table 3) Only one-third of CEA authors modeled increased mortality following hip fractures (n = 6, 33%). A few authors also incorporated increased mortality following adverse events associated with tamoxifen, including thromboembolism (n = 4, 22%), endometrial cancer (n = 4, 22%), and cardiovascular events (n = 3, 17%).

### Handling of Methodological Uncertainty

Few CEAs performed sub-group or scenario analyses to address patient heterogeneity related to older women (n = 6, 33%), women at low risk of breast cancer recurrence (n = 2, 11%), and women with multiple co-morbid diseases (n = 1, 6%). The majority of CEAs conducted the analyses from the perspective of the health care system as payer, accounting for the costs attributable to provider organizations such as government payers or other health insurers. The perspective excludes patient costs such as lost productivity and out of pocket health expenditures which may affect CEA results. The time horizon modeled ranged from 20 years to lifetime. Despite the fact that 17 of 18 studies modeled breast cancer recurrence risk beyond the follow-up time from trial data, a large proportion did not assess the impact of uncertainty arising from extrapolating beyond the trial data. (n = 6, 35%) Discount rates were reported in 17 of 18 studies and ranged from 1.5% to 6%. Five studies (25%) did not vary the discount rate in sensitivity analysis.

Detailed information on handling of structural and methodological uncertainty is available in Table S4.

Only two of the studies mentioned limitations associated with external validity of CEA findings based on RCT data, but discussion of this limitation was not extensive in either case. (Table S4).

## Discussion

Our review identified 18 published CEAs that compared AIs with tamoxifen for the first line treatment of early breast cancer in post-menopausal women. We found a lack of sensitivity and sub-group analyses that could limit the relevance of the study findings. The CEA studies assumed that observed benefits of AI in terms of DFS observed in individual RCTs would lead to improved OS. Subsequent meta-analysis of RCTs and longer term follow up provide no evidence of an OS benefit with five years of AI. [12], [13] Although increased rates of adverse events, particularly fractures, were found with the use of AI, many of the published CEAs did not adequately investigate the impact of these adverse events. Potential heterogeneity of the results across important sub-groups such as older women or women at risk for adverse events was often ignored in the published studies. The limitations of the existing CEA studies of AI reduce their relevance to policy making and assessment of value for money in cancer care.

The ICERs from the 18 published analyses appear to be generally consistent with other cost-effectiveness analyses of breast cancer related interventions. A systematic review identified 89 cost-effectiveness analyses for breast cancer related interventions with a median ICUR of $27 K (2008 USD $/QALY). [50] All ICERs from the published analyses were less than $100 K (2010 USD) per life years and the majority less than $50 K (2010 USD) per life year. All ICURs with the exception of the analysis by Gil et al comparing anastrazole to tamoxifen were under the threshold of $100 K (2010 USD) per QALY and the majority under $50 K per QALY. Even though ICERs and ICURs varied somewhat amongst the analyses, the publications conveyed a consistent message. The 18 studies generally indicate that adopting aromatase inhibitors as first line therapy is good value for money when compared to tamoxifen since few of the studies exceeded commonly accepted thresholds. The validity of the assertion that aromatase inhibitors are a cost-effective alternative to tamoxifen rests on the quality and thoroughness of the analyses. Our critique of these analyses in light of best practices for assessing uncertainty raises concerns about validity and signals that the ICERs and ICURs may be underestimates of the cost-effectiveness of aromatase inhibitors for women with early stage breast cancer. The assumption that aromatase inhibitors lengthen survival is a major component of the published analyses. If aromatase inhibitors do not improve survival, then the cost-effectiveness values are underestimated and incorporating more realistic assumptions may raise cost-effectiveness ratios. Our critique of these analyses implies that health policy related to aromatase inhibitors should be revisited.

Our study provides the most recent and comprehensive view of CEAs addressing AIs in the first-line setting. Compared to other systematic reviews we employ no language restrictions. [51], [52] We also systematically assess important potential sources of bias in CEA. Our analysis also compares predictions of model-based CEAs for AIs to clinical data. Following the release of preliminary results from the ATAC and BIG trials, clinicians widely expected AI reductions in breast cancer recurrence to translate into survival gains, much as tamoxifen reduced breast cancer recurrence risk and subsequently increased survival relative to no hormonal therapy. [53] Published CEAs universally predicted increased survival for AIs but reports from the ATAC and BIG trials demonstrated no significant differences in overall survival. [54]–[57] Pooled analysis of data from both trials indicated that at 5 years the overall difference in mortality between patients treated with AIs and those treated with tamoxifen was 0·8% and this decreased to 0·2% at 8 years. [13] In neither case was the difference in OS significant. Even with longer follow-up a divergence in survival curves between AI and tamoxifen treated patients is unlikely to reach the levels estimated by the CEA models. Disparities between long-term outcomes and CEA predictions relate to methods for extrapolating trial data into the future, and assumptions about the impact of adverse events on mortality. Leading oncologists correctly note that with no real survival benefit, the ICER - the incremental cost per life year or quality adjusted life year - associated with AIs in the first-line setting is much higher than published analyses suggest. [17], [18] Indeed, clinical opinion leaders have advocated for switching from tamoxifen to an aromatase inhibitor after two to three years, a strategy that clinical trials indicate may confer a survival benefit compared to five years of tamoxifen. [58], [59]The method for translating outcomes such as disease free survival into survival gains has significant implications on CEA estimates and resulting policy guidance.

Our paper adds to a growing body of literature elucidating the mechanisms through which CEAs can produce potentially misleading results. [60], [61] For example, Polyzos et al demonstrated that industry-sponsored CEAs assessing cervical cancer screening were more likely to exclude data sources presenting favourable results for existing technologies. [60], [61] We can consider our results in light of approaches for addressing parameter uncertainty, structural uncertainty, and methodological uncertainty in CEA modeling. Despite significant parameter uncertainty about the true value of the relative risk of breast cancer recurrence for AIs compared to tamoxifen, 44% of analyses we identified did not vary this critical parameter in even one-way sensitivity analysis. Uncertainty arising from the impact of adverse events can be addressed by adapting the model structure to model adverse events and resulting outcomes. Published CEAs of aromatase inhibitors did not adequately explore structural uncertainty. Adverse events were inadequately modeled, and the potential for increased mortality resulting from adverse events largely overlooked. Methodological uncertainty was also inadequately addressed. Thirty-five percent of studies did not test methodological approaches for extrapolating short-term outcomes from RCTs to a longer time horizon. Twenty-eight percent of studies did not vary the discount rates. Our findings are consistent with a recent systematic review of all published CEAs demonstrating that many authors do a poor job of accounting for uncertainty. [26].

Our analysis has some limitations. Jang et al demonstrated that industry sponsored CEAs were significantly more likely than CEAs conducted by independent academic centres to reach favourable conclusions about the cost-effectiveness of AIs. [52] It was beyond the scope of our analysis to quantify the impact of industry sponsorship on methodological choices and CEA conclusions, even though association between industry-sponsorship and favourable CEA findings is well established. [62]–[64] We focused only on AIs in the first-line setting, and thus had limited statistical power to detect differences. However, we demonstrate how favourable results from industry-sponsored clinical studies may propagate through CEAs when study results are incorporated with no adjustment for real world settings. [65] Our analysis did not include a search of the grey literature, and thus we may have missed some CEAs published by government agencies. Some authors published multiple models identified by our systematic review and thus study data are not strictly independent. However, in each case of multiple models, authors addressed different jurisdictions, thus the question of whether guidance is policy relevant is important for each. The small number of studies precludes analysis of variance in CEA estimates arising from differences in data sources, study perspective, cost categories, time horizon, discount rates, adverse events or utility measurement techniques. Furthermore, authors provided insufficient information on factors like cost categories and utility measurement techniques to allow for this kind of analysis. Finally, it was beyond the scope of our study to summarize the results of sensitivity analyses as authors report sensitivity analyses in different ways, employing different criteria and different thresholds for identifying important variables using sensitivity analysis.

### Conclusion

Published CEAs comparing AIs to tamoxifen inadequately investigate uncertainty to overcome the limitations of translating RCT findings to real-world practice, potentially leading to suboptimal guidance for clinical and health policy decision-making. The implications of these findings extend beyond hormonal therapies for early stage breast cancer to other cancer therapies and drug therapies in general. Care must be taken when interpreting CEAs based on RCTs which employ surrogate endpoints with populations that differ from the real-world population. [2] Even when RCT data provides the best evidence on efficacy, CEA authors are encouraged to utilize the potential of CEAs to overcome limitations of RCT-based guidance. Such an approach would leverage existing knowledge on the natural history of disease and consider uncertainty to better inform adoption of new cancer therapies.

## Supporting Information

Table S1 Study characteristics.
(DOC)

Table S2 Neumann appraisal.
(PDF)

Table S3 Data sources and handling of parameter uncertainty.
(DOC)

Table S4 Handling of structural and methodological uncertainty.
(DOC)

Appendix S1 **Terminology used to search literature databases.**
(DOC)

Appendix S2 **References to the online supplemental documents.**
(DOC)

## References

[1] Smith TJ, Hillner BE (2011) Bending the Cost Curve in Cancer Care. New Engl J Med 364: 2060–2065. 21612477 10.1056/NEJMsb1013826 PMC4042405

[2] Sullivan R, Peppercorn J, Sikora K, Zalcberg J, Meropol NJ, et al (2011) Delivering affordable cancer care in high-income countries. Lancet Oncol 12: 933–980. 21958503 10.1016/S1470-2045(11)70141-3

[3] Drummond M, O’Brien B, Stoddart GL, Torrance GW (2005) Methods for the Economic Evaluation of Health Care Programmes. New York: Oxford University Press Inc. 379 p.

[4] Laupacis A, Feeny D, Detsky AS, Tugwell PX (1992) How attractive does a new technology have to be to warrant adoption and utilization? Tentative guidelines for using clinical and economic evaluations. Can Med Assoc J 146: 473–481. 1306034 PMC1488412

[5] Shiroiwa T, Sung Y-K, Fukuda T, Lang H-C, Bae S-C, et al (2010) International survey on willingness-to-pay (WTP) for one additional QALY gained: what is the threshold of cost effectiveness? Health Econ 19: 422–437. 19382128 10.1002/hec.1481

[6] Neumann PJ, Goldie SJ, Weinstein MC (2000) Preference-based measures in economic evaluation in health care. Annu Rev Public Health 21: 587–611. 10884966 10.1146/annurev.publhealth.21.1.587

[7] Torrance GW (1987) Utility approach to measuring health-related quality of life. J Chron Dis 40: 593–603. 3298297 10.1016/0021-9681(87)90019-1

[8] Roberts M, Russell LB, Paltiel AD, Chambers M, McEwan P, et al (2012) Conceptualizing a model: a report of the ISPOR-SMDM Modeling Good Research Practices Task Force-2. Med Decis Making 32: 678–689. 22990083 10.1177/0272989X12454941

[9] Briggs AH, Weinstein MC, Fenwick EA, Karnon J, Sculpher MJ, et al (2012) Model parameter estimation and uncertainty analysis: a report of the ISPOR-SMDM Modeling Good Research Practices Task Force Working Group-6. Med Decis Making 32: 722–732. 22990087 10.1177/0272989X12458348

[10] ATAC Trialists’ Group, Forbes JF, Cuzick J, Buzdar A, Howell A, et al (2008) Effect of anastrozole and tamoxifen as adjuvant treatment for early-stage breast cancer: 100-month analysis of the ATAC trial. Lancet Oncol 9: 45–53. 18083636 10.1016/S1470-2045(07)70385-6

[11] BIG 1–98 Collaborative Group, Mouridsen H, Giobbie-Hurder A, Goldhirsch A, Thurlimann B, et al (2009) Letrozole therapy alone or in sequence with tamoxifen in women with breast cancer. New Engl J Med 361: 766–776. 19692688 10.1056/NEJMoa0810818 PMC2921823

[12] Amir E, Seruga B, Niraula S, Carlsson L, Ocaña A (2011) Toxicity of Adjuvant Endocrine Therapy in Postmenopausal Breast Cancer Patients: A Systematic Review and Meta-analysis. J Natl Cancer I 103: 1299–1309. 10.1093/jnci/djr242 21743022

[13] Dowsett M, Cuzick J, Ingle J, Coates A, Forbes J, et al (2010) Meta-analysis of breast cancer outcomes in adjuvant trials of aromatase inhibitors versus tamoxifen. J Clin Oncol 28: 509–518. 19949017 10.1200/JCO.2009.23.1274

[14] Pezo RC, Seruga B, Krzyzanowska MK, Bedard P (2011) Quality of safety reporting in oncology-randomized controlled trials (RCTs). J Clin Oncol 29.

[15] Neuner JM, Yen TW, Sparapani RA, Laud PW, Nattinger AB (2011) Fracture risk and adjuvant hormonal therapy among a population-based cohort of older female breast cancer patients. Osteoporosis Int 22: 2847–2855. 10.1007/s00198-010-1493-x PMC3166362 21170643

[16] Rabaglio M, Sun Z, Price KN, Castiglione-Gertsch M, Hawle H, et al (2009) Bone fractures among postmenopausal patients with endocrine-responsive early breast cancer treated with 5 years of letrozole or tamoxifen in the BIG 1–98 trial. Ann Oncol 20: 1489–1498. 19474112 10.1093/annonc/mdp033 PMC2731016

[17] Seruga B, Ocana A, Niraula S, Amir E (2010) Absolute benefits of aromatase inhibitors in adjuvant treatment of breast cancer: should we know more? J Clin Oncol 28: e346–347; author reply e348. 10.1200/JCO.2010.28.3564 20458046

[18] Seruga B, Tannock IF (2009) Up-front use of aromatase inhibitors as adjuvant therapy for breast cancer: the emperor has no clothes. J Clin Oncol 27: 840–842. 19139426 10.1200/JCO.2008.19.5594

[19] Google Translate API. Available: http://www.webcitation.org/5z1B8xoUj. Accessed 2013 Apr 8.

[20] Neumann PJ, Stone PW, Chapman RH, Sandberg EA, Bell CM (2000) The quality of reporting in published cost-utility analyses, 1976–1997.[see comment]. Ann Intern Med 132: 964–972. 10858180 10.7326/0003-4819-132-12-200006200-00007

[21] International Bank for Reconstruction and Development/The World Bank (2008) Global Purchasing Power Parities and Real Expenditures - 2005 International Comparison Program. Available: http://siteresources.worldbank.org/ICPINT/Resources/icp-final.pdf. Accessed 2013 Apr 8.

[22] The World Bank (2005) International Comparison Program. Available: http://data.worldbank.org/data-catalog. Accessed 2013 Apr 8.

[23] Sroczynski G, Esteban E, Conrads-Frank A, Schwarzer R, Muhlberger N, et al (2009) Long-term effectiveness and cost-effectiveness of screening for hepatitis C virus infection. Eur J Public Health 19: 245–253. 19196737 10.1093/eurpub/ckp001

[24] Sroczynski G, Esteban E, Conrads-Frank A, Schwarzer R, Muhlberger N, et al (2010) Long-term effectiveness and cost-effectiveness of antiviral treatment in hepatitis C. J Viral Hepat. 17: 34–50. 10.1111/j.1365-2893.2009.01147.x 19656290

[25] Bureau of Labor Statistics: United States Department of Labor. (2010) CPI for All Urban Consumers (CPI-U) 1982–84 = 100 (Unadjusted) - CUUR0000SA0. Available: http://146.142.4.24/cgi-bin/surveymost?bls Accessed 2013 Apr 8.

[26] Jain R, Grabner M, Onukwugha E (2011) Sensitivity analysis in cost-effectiveness studies: from guidelines to practice. Pharmacoeconomics 29: 297–314. 21395350 10.2165/11584630-000000000-00000

[27] Briggs AH, Gray AM (1999) Handling uncertainty in economic evaluations of healthcare interventions. Brit Med J 319: 635–638. 10473486 10.1136/bmj.319.7210.635 PMC1116497

[28] Coyle D, Cranney A, Lee KM, Welch V, Tugwell P (2001) Cost effectiveness of nasal calcitonin in postmenopausal women: use of Cochrane Collaboration methods for meta-analysis within economic evaluation. Pharmacoeconomics 19: 565–575. 11465301 10.2165/00019053-200119050-00010

[29] Sculpher M (2008) Subgroups and heterogeneity in cost-effectiveness analysis. Pharmacoeconomics 26: 799–806. 18767899 10.2165/00019053-200826090-00009

[30] Ginnelly L, Claxton K, Sculpher MJ, Golder S (2005) Using value of information analysis to inform publicly funded research priorities. Appl Health Econ Health Policy 4: 37–46. 16076237 10.2165/00148365-200504010-00006

[31] Haentjens P, Magaziner J, Colon-Emeric CS, Vanderschueren D, Milisen K, et al (2010) Meta-analysis: excess mortality after hip fracture among older women and men. Ann Intern Med 152: 380–390. 20231569 10.1059/0003-4819-152-6-201003160-00008 PMC3010729

[32] Delea TE, El-Ouagari K, Karnon J, Sofrygin O, Delea TE, et al (2008) Cost-effectiveness of letrozole versus tamoxifen as initial adjuvant therapy in postmenopausal women with hormone-receptor positive early breast cancer from a Canadian perspective. Breast Cancer Res Treat 108: 375–387. 17653859 10.1007/s10549-007-9607-7

[33] Delea TE, Karnon J, Sofrygin O, Thomas SK, Papo NL, et al (2007) Cost-effectiveness of letrozole versus tamoxifen as initial adjuvant therapy in hormone receptor-positive postmenopausal women with early-stage breast cancer. Clin Breast Cancer 7: 608–618. 17592673 10.3816/CBC.2007.n.018

[34] Fonseca M, Araujo GT, Saad ED, Fonseca M, Araujo GTB, et al (2009) Cost-effectiveness of anastrozole, in comparison with tamoxifen, in the adjuvant treatment of early breast cancer in Brazil. Rev Assoc Med Bras 55: 410–415. 19750307 10.1590/s0104-42302009000400015

[35] Gamboa O, Diaz S, Chicaiza L, Garcia M (2010) [Cost-benefit analysis of anastrazol and tamoxifen in adjuvant treatment of hormone receptor-positive, post-menopausal breast cancer]. [Spanish]. Biomedica 30: 46–55. 20890549

[36] Gil JM, Rubio-Terres C, Del Castillo A, Gonzalez P, Canorea F, et al (2006) Pharmacoeconomic analysis of adjuvant therapy with exemestane, anastrozole, letrozole or tamoxifen in postmenopausal women with operable and estrogen receptor-positive breast cancer. Clini Trans Oncol 8: 339–348. 10.1007/s12094-006-0180-z 16760009

[37] Hillner BE (2004) Benefit and projected cost-effectiveness of anastrozole versus tamoxifen as initial adjuvant therapy for patients with early-stage estrogen receptor-positive breast cancer. Cancer 101: 1311–1322. 15368322 10.1002/cncr.20492

[38] Hind D, Ward S, De Nigris E, Simpson E, Carroll C, et al.. (2007) Hormonal therapies for early breast cancer: systematic review and economic evaluation. Health Technol Assess 11: iii–iv. 10.3310/hta11260 17610808

[39] Karnon J, Delea T, Barghout V, Karnon J, Delea T, et al (2008) Cost utility analysis of early adjuvant letrozole or anastrozole versus tamoxifen in postmenopausal women with early invasive breast cancer: the UK perspective. Eur J Health Econ 9: 171–183. 17602251 10.1007/s10198-007-0058-1

[40] Lazzaro C (2007) Cost-utility analysis of anastrozole versus tamoxifen for adjuvant treatment in postmenopausal women with early breast cancer. [Italian]. PharmacoEconomics - Italian Research Articles 9: 31–43.

[41] Lee HJ, Lee TJ, Yang BM, Min J (2010) Cost-effectiveness analysis of adjuvant hormonal treatments for women with postmenopausal hormone-receptor positive early breast cancer in the Korean context. J Breast Canc 13: 286–298.

[42] Locker GY, Mansel R, Cella D, Dobrez D, Sorensen S, et al (2007) Cost-effectiveness analysis of anastrozole versus tamoxifen as primary adjuvant therapy for postmenopausal women with early breast cancer: a US healthcare system perspective. The 5-year completed treatment analysis of the ATAC (‘Arimidex’, Tamoxifen Alone or in Combination) trial. Breast Cancer Res Treat 106: 229–238. 17245540 10.1007/s10549-006-9483-6

[43] Lux MP, Wockel A, Benedict A, Buchholz S, Kreif N, et al (2010) Cost-effectiveness analysis of anastrozole versus tamoxifen in adjuvant therapy for early-stage breast cancer - a health-economic analysis based on the 100-month analysis of the ATAC trial and the German health system. Onkologie 33: 155–166. 20389141 10.1159/000286233

[44] Mansel R, Locker G, Fallowfield L, Benedict A, Jones D, et al (2007) Cost-effectiveness analysis of anastrozole vs tamoxifen in adjuvant therapy for early stage breast cancer in the United Kingdom: the 5-year completed treatment analysis of the ATAC (‘Arimidex’, Tamoxifen alone or in combination) trial. Brit J Cancer 97: 152–161. 17622238 10.1038/sj.bjc.6603804 PMC2360294

[45] Moeremans K, Annemans L (2006) Cost-effectiveness of anastrozole compared to tamoxifen in hormone receptor-positive early breast cancer. Analysis based on the ATAC trial. Int J Gynecol Cancer 16 Suppl 2576–578. 17010076 10.1111/j.1525-1438.2006.00699.x

[46] Rocchi A, Verma S (2006) Anastrozole is cost-effective vs tamoxifen as initial adjuvant therapy in early breast cancer: Canadian perspectives on the ATAC completed-treatment analysis. Support Care Cancer 14: 917–927. 16596419 10.1007/s00520-006-0035-8

[47] Sasse AD, Sasse EC (2009) [Cost-effectiveness analysis of adjuvant anastrozol in post-menopausal women with breast cancer]. [Portuguese]. Rev Assoc Med Bras 55: 535–540. 19918652 10.1590/s0104-42302009000500015

[48] Skedgel C, Rayson D, Dewar R, Younis T, Skedgel C, et al (2007) Cost-utility of adjuvant hormone therapies with aromatase inhibitors in post-menopausal women with breast cancer: upfront anastrozole, sequential tamoxifen-exemestane and extended tamoxifen-letrozole. Breast 16: 252–261. 17207623 10.1016/j.breast.2006.12.002

[49] Skedgel C, Rayson D, Dewar R, Younis T, Skedgel C, et al (2007) Cost-utility of adjuvant hormone therapies for breast cancer in post-menopausal women: sequential tamoxifen-exemestane and upfront anastrozole. Breast Cancer Res Treat 101: 325–333. 16897433 10.1007/s10549-006-9299-4

[50] Greenberg D, Earle C, Fang CH, Eldar-Lissai A, Neumann PJ (2010) When is cancer care cost-effective? A systematic overview of cost-utility analyses in oncology. J Natl Cancer Inst 102: 82–88. 20056956 10.1093/jnci/djp472 PMC2808348

[51] Annemans L, Annemans L (2008) Methodological issues in evaluating cost effectiveness of adjuvant aromatase inhibitors in early breast cancer: a need for improved modelling to aid decision making. Pharmacoeconomics 26: 409–423. 18429657 10.2165/00019053-200826050-00005

[52] Jang S, Chae YK, Haddad T, Majhail NS (2010) Conflict of interest in economic analyses of aromatase inhibitors in breast cancer: a systematic review. Breast Cancer Res Treat 121: 273–279. 20352486 10.1007/s10549-010-0870-7

[53] Early Breast Cancer Trialists’ Collaborative Group (EBCTCG) (2005) Effects of chemotherapy and hormonal therapy for early breast cancer on recurrence and 15-year survival: an overview of the randomised trials. Lancet 365: 1687–1717. 15894097 10.1016/S0140-6736(05)66544-0

[54] ATAC Trialists’ Group (2005) Results of the ATAC (Arimidex, Tamoxifen, Alone or in Combination) trial after completion of 5 years’ adjuvant treatment for breast cancer. Lancet 365: 60–62. 15639680 10.1016/S0140-6736(04)17666-6

[55] B. I. G. Collaborative Group (2009) Letrozole Therapy Alone or in Sequence with Tamoxifen in Women with Breast Cancer. New Engl J Med 361: 766–776. 19692688 10.1056/NEJMoa0810818 PMC2921823

[56] Coates AS, Keshaviah A, Thürlimann B, Mouridsen H, Mauriac L, et al (2007) Five Years of Letrozole Compared With Tamoxifen As Initial Adjuvant Therapy for Postmenopausal Women With Endocrine-Responsive Early Breast Cancer: Update of Study BIG 1–98. J Clin Oncol 25: 486–492. 17200148 10.1200/JCO.2006.08.8617

[57] Cuzick J, Sestak I, Baum M, Buzdar A, Howell A, et al (2010) Effect of anastrozole and tamoxifen as adjuvant treatment for early-stage breast cancer: 10-year analysis of the ATAC trial. Lancet Oncol 11: 1135–1141. 21087898 10.1016/S1470-2045(10)70257-6

[58] Jassem J (2008) Intergroup Exemestane Study mature analysis: overall survival data. Anti-Cancer Drugs 19 Suppl 1S3–7. 10.1097/01.cad.0000277608.23376.bc 18340242

[59] Jonat W, Gnant M, Boccardo F, Kaufmann M, Rubagotti A, et al (2006) Effectiveness of switching from adjuvant tamoxifen to anastrozole in postmenopausal women with hormone-sensitive early-stage breast cancer: a meta-analysis. Lancet Oncol 7: 991–996. 17138220 10.1016/S1470-2045(06)70948-2

[60] Chauhan D, Miners AH, Fischer AJ (2007) Exploration of the difference in results of economic submissions to the National Institute of Clinical Excellence by manufacturers and assessment groups. Int J Technol Assess 23: 96–100. 10.1017/S0266462307051628 17234022

[61] Polyzos NP, Valachis A, Mauri D, Ioannidis JP (2011) Industry involvement and baseline assumptions of cost-effectiveness analyses: diagnostic accuracy of the Papanicolaou test. Can Med Assoc J 183: E337–343. 21402681 10.1503/cmaj.101506 PMC3071415

[62] Bell CM, Urbach DR, Ray JG, Bayoumi A, Rosen AB, et al (2006) Bias in published cost effectiveness studies: systematic review. Brit Med J 332: 699–703. 16495332 10.1136/bmj.38737.607558.80 PMC1410902

[63] Garattini L, Koleva D, Casadei G (2010) Modeling in pharmacoeconomic studies: funding sources and outcomes. Int J Technol Assess 26: 330–333. 10.1017/S0266462310000322 20584363

[64] John-Baptiste A, Bell C (2011) Industry sponsored bias in cost effectiveness analyses. Brit Med J 341: c5350. 10.1136/bmj.c5350 20943724

[65] John-Baptiste AA, Bell C (2011) A glimpse into the black box of cost-effectiveness analyses. Can Med Assoc J 183: E307–308. 21402688 10.1503/cmaj.110384 PMC3071404

