# Supplementary material for: A Systematic Review and Methodological Evaluation of Published Cost-Effectiveness Analyses of Aromatase Inhibitors versus Tamoxifen in Early Stage Breast Cancer
Source: PLoS One. 2013 May 6;8(5):e62614. doi: 10.1371/journal.pone.0062614 (PMC3646035; doi:10.1371/journal.pone.0062614)
Supplement: Table S1 — Study characteristics. (DOC) [file pone.0062614.s001.doc]

**Table S1: Study characteristics**

| **No.** | **Paper** | **Publication Year** | **Country** | **Aromatase Inhibitor(s)** | **Outcomes** | **Perspective** | **Type of Model** | **Sponsorship** | **Time Horizon** | **Discount Rates** |
| --- | --- | --- | --- | --- | --- | --- | --- | --- | --- | --- |
| 1 | Delea^1^ | 2007 | United States | Letrozole | Life years, QALYs | Health Care Payer | Markov Cohort | Industry | 30 years | 3% |
| 2 | Delea^2^ | 2008 | Canada | Letrozole | Life years, QALYs | Health Care Payer | Markov Cohort | Industry | 30 years | 5% |
| 3 | Fonseca^3^ | 2009 | Brazil | Anastrazole | Life years | Health Care Payer | Markov Cohort | Industry | Lifetime | Cost - 3%, Life years - 1.5% |
| 4 | Gamboa^4^ | 2010 | Colombia | Anastrazole | Life years | Health Care Payer | Markov Cohort | Government agency | 30 years | 3% |
| 5 | Gil^5^ | 2006 | Spain | Anastrazole | Life years, QALYs | Health Care Payer | Markov Cohort | Industry | 20 years | 3.50% |
| 6 | Hillner^6^ | 2004 | United States | Anastrazole | Life years, QALYs | Health Care Payer | Markov Cohort | Government agency | 20 years | 3% |
| 7 | Hind^7^ | 2007 | United Kingdom | Anastrazole, Letrozole | Life years, QALYs | Health Care Payer | Markov Cohort | Government agency | 35 years | Cost - 6%, Life Years/QALYs - 1.5% |
| 8 | Karnon^8^ | 2008 | United Kingdom | Anastrazole, Letrozole | Life years, QALYs | Health Care Payer | Markov Cohort | Industry | 50 years | 3.50% |
| 9 | Lazzaro^9^ | 2007 | Italy | Anastrazole | QALYs | Health Care Payer | Unclear | Industry | 68 months | 3% |
| 10 | Lee^10^ | 2010 | Korea | Anastrazole, Letrozole | QALYs | Societal | Markov Cohort | Not Stated | 35 years | 5% |
| 11 | Locker^11^ | 2007 | United States | Anastrazole | Life years, QALYs | Health Care Payer | Markov Cohort | Industry | 25 years | 3% |
| 12 | Lux^12^ | 2010 | Germany | Anastrazole | QALYs | Health Care Payer | Markov Cohort | Industry | 25 years | 3% |
| 13 | Mansel^13^ | 2007 | United Kingdom | Anastrazole | Life years, QALYs | Health Care Payer | Markov Cohort | Industry | 25 years | 3.50% |
| 14 | Moeremans^14^ | 2006 | Belgium | Anastrazole | Life years, QALYs | Health Care Payer | Markov Cohort | Not Stated | 20 years | Not Reported |
| 15 | Rocchi^15^ | 2006 | Canada | Anastrazole | Life years, QALYs | Health Care Payer | Markov Cohort | Industry | Lifetime | 5% |
| 16 | Sasse^16^ | 2009 | Brazil | Anastrazole | QALYs | Multiple Perspectives | Markov Cohort | Not Stated | 25 years | 3% |
| 17 | Skedgel^17^ | 2007 | Canada | Anastrazole | QALYs | Health Care Payer | Markov Cohort | Industry, Charitable Foundation | 20 years | 3% |
| 18 | Skedgel^18^ | 2007 | Belgium | Anastrazole | QALYs | Health Care Payer | Markov Cohort | Industry | 20 years | 3% |

QALYs indicates quality adjusted life years
