# Supplementary material for: A Systematic Review and Methodological Evaluation of Published Cost-Effectiveness Analyses of Aromatase Inhibitors versus Tamoxifen in Early Stage Breast Cancer
Source: PLoS One. 2013 May 6;8(5):e62614. doi: 10.1371/journal.pone.0062614 (PMC3646035; doi:10.1371/journal.pone.0062614)
Supplement: Table S2 — Neumann appraisal. (PDF) [file pone.0062614.s002.pdf]

Table S2: Neumann appraisal[19]

	Delea[1]	*Delea[2]*	*Fonseca[3]*	*Gamboa[4]*	*Gil[5]*	*Hillner[6]*	*Hind[7]*	*Karnon[8]*	*Lazzaro[9]*	*Lee[10]*	*Locker[11]*	*Lux[12]*	*Mansel[13]*	*Moeremans[14]*	*Rocchi[15]*	*Sasse[16]*	*Skedgel[17]*	*Skedgel[18]*
**A Framing**																		
**A.1. Funding source disclosed?**	Y	Y	Y	Y	Y	Y	Y	Y	Y	N	Y	Y	Y	N	Y	N	Y	Y
**A.1.1. Specify funding source (1=industry, 2=academic/non-profit, 3=other)**	1	1	1	2	1	2	2	1	1		1	1	1		1		3	1
**A.2 .Study perspective clearly stated?**	Y	Y	Y	Y	Y	Y	Y	Y	Y	Y	Y	Y	Y	Y	Y	Y	Y	Y
**A.3. Modeling assumptions listed?**	Y	Y	Y	Y	Y	Y	Y	Y	N	Y	Y	Y	Y	Y	Y	Y	Y	Y
**A.4. Diagram of model or event pathway provided?**	Y	Y	N	Y	Y	Y	Y	Y	N	Y	Y	Y	Y	Y	Y	Y	Y	Y
**A.5. Discount rate for future costs and QALYs reported?**	Y	Y	Y	Y	Y	Y	Y	Y	Y	Y	Y	Y	Y	N	Y	Y	Y	Y
**B Reporting of costs**																		
**B.1. Were net costs reported?**	Y	Y	Y	Y	Y	Y	Y	Y	Y	Y	Y	Y	Y	Y	Y	Y	Y	Y
**B.2. Was the source of valuation for all cost items reported?**	Y	Y	Y	Y	Y	Y	Y	Y	Y	Y	Y	Y	Y	N	Y	Y	Y	Y
**B.3. Did the authors clearly state the year of monetary units?**	Y	Y	Y	Y	Y	N	Y	Y	Y	Y	Y	Y	Y	N	Y	Y	Y	Y
**B.4. Were preference weights reported?**	Y	Y	N/A	N/A	Y	Y	Y	Y	Y	N	Y	Y	Y	Y	Y	Y	Y	Y
**B.5. Was the preference measurement technique reported?**	Y	Y	N/A	N/A	N	N	Y	Y	N	N	Y	Y	Y	N	N	N	N	N
**B.6. Was the source of preferences listed?**	Y	Y	N/A	N/A	Y	Y	Y	Y	Y	Y	Y	Y	Y	Y	Y	Y	Y	Y
**C Reporting of results**																		
**C.1. Were incremental analyses appropriately reported?**	Y	Y	Y	Y	Y	Y	Y	Y	Y	Y	Y	Y	Y	Y	Y	Y	Y	Y
**C.2. Were sensitivity analyses reported?**	Y	Y	Y	Y	Y	Y	Y	Y	Y	Y	Y	Y	Y	N	Y	N	Y	Y
**C.2.1. for costs?**	Y	Y	Y	Y	Y	Y	Y	Y	N	Y	Y	Y	Y	N	Y	N	Y	Y
**C.2.2. for preference weights?**	Y	Y	N/A	N/A	N	N	N	Y	N	N	N	Y	Y	N	N	N	Y	Y
**C.2.3. for estimates of effectiveness?**	Y	Y	N	N	N	Y	Y	Y	N	Y	Y	Y	N	N	Y	N	Y	Y
**C.2.4. for discount rate?**	Y	Y	N	Y	Y	N	Y	Y	Y	Y	N	Y	Y	N	Y	N	Y	Y
**D Discussion**																		
**D.1. Were study limitations discussed?**	Y	Y	N	Y	Y	N	Y	Y	Y	Y	Y	Y	Y	N	Y	Y	Y	Y
**D.2. Were results compared with those of related cost-utility analyses?**	Y	Y	Y	Y	Y	N	Y	Y	N	N	Y	Y	Y	N	Y	Y	Y	Y
**D.3. Were the ethical implications discussed?**	N	N	N	N	N	N	N	N	N	N	N	N	N	N	N	N	N	N
**E Overall Assessment**	5.25	5.25	1	1.5	2.5	4.25	5.25	5.25	1	3	4.25	4.25	4.25	1.5	4.75	1	4	4
