# Supplementary material for: A Systematic Review and Methodological Evaluation of Published Cost-Effectiveness Analyses of Aromatase Inhibitors versus Tamoxifen in Early Stage Breast Cancer
Source: PLoS One. 2013 May 6;8(5):e62614. doi: 10.1371/journal.pone.0062614 (PMC3646035; doi:10.1371/journal.pone.0062614)
Supplement: Table S3 — Data sources and handling of parameter uncertainty. (DOC) [file pone.0062614.s003.doc]

**Table S3: Data sources and handling of parameter uncertainty**

| **No.** | **Author** | **Source of information on recurrence rates** | **Sensitivity analysis on the risk of breast cancer recurrence?** | **Source of information on adverse events** | **Sensitivity analysis on …** | | | | | **PSA** | **VOI** |
| --- | --- | --- | --- | --- | --- | --- | --- | --- | --- | --- | --- |
|  |  |  |  |  | **Fracture** | **Cardio-vascular events** | **Stroke** | **Thrombo-embolism** | **Endo-metrial cancer** |  |  |
| 1 | Delea^1^ | Single RCT | Y | Combination^a^ | Y | Y | N | Y | Y | Y | N |
| 2 | Delea^2^ | Single RCT | Y | Combination^a^ | Y | Y | N | Y | Y | Y | N |
| 3 | Fonseca^3^ | Single RCT | N | Single RCT | N | N | N | N | N | Y | N |
| 4 | Gamboa^4^ | Single RCT | N | Other^b^ | N | N | N | Y | Y | Y | N |
| 5 | Gil^5^ | Single RCT | N | Single RCT | N | N | N | N | N | N | N |
| 6 | Hillner^6^ | Single RCT | Y | Combination^a^ | Y | N | N | N | N | N | N |
| 7 | Hind^7^ | Single RCT | N | Single RCT | Y | N | N | N | N | Y | N |
| 8 | Karnon^8^ | Combination^a^ | Y | Combination^a^ | N | N | N | N | N | Y | N |
| 9 | Lazzaro^9^ | Single RCT | N | Single RCT | N | N | N | N | N | Y | N |
| 10 | Lee^10^ | Combination^a^ | N | Combination^a^ | Y | Y | N | Y | Y | N | N |
| 11 | Locker^11^ | Single RCT | Y | Combination^a^ | Y | N | N | N | N | Y | N |
| 12 | Lux^12^ | Single RCT | Y | Combination^a^ | N | N | N | N | N | Y | N |
| 13 | Mansel^13^ | Single RCT | Y | Combination^a^ | N | N | N | N | N | Y | N |
| 14 | Moeremans^14^ | Single RCT | N | Single RCT | N | N | N | N | N | N | N |
| 15 | Rocchi^15^ | Single RCT | Y | Single RCT | N | N | N | N | N | Y | N |
| 16 | Sasse^16^ | Single RCT | N | Single RCT | N | N | N | N | N | N | N |
| 17 | Skedgel^17^ | Combination^a^ | Y | Single RCT | N | N | N | N | N | N | N |
| 18 | Skedgel^18^ | Combination^a^ | Y | Single RCT | N | N | N | N | N | N | N |

a Authors combined observational data or a risk model with RCT data

b Authors cited multiple data sources for information on harms but it was unclear what information was incorporated into the model.

PSA – Probabilistic Sensitivity Analysis

VOI – Value of Information
