# Supplementary material for: A Systematic Review and Methodological Evaluation of Published Cost-Effectiveness Analyses of Aromatase Inhibitors versus Tamoxifen in Early Stage Breast Cancer
Source: PLoS One. 2013 May 6;8(5):e62614. doi: 10.1371/journal.pone.0062614 (PMC3646035; doi:10.1371/journal.pone.0062614)
Supplement: Table S4 — Handling of structural and methodological uncertainty. (DOC) [file pone.0062614.s004.doc]

**Table S4: Handling of structural and methodological uncertainty**

| **No.** | **Author** | **Increased mortality following any adverse event?** | **Did the analysis address the following sub-groups?** | | | | | | | |  |  |
| --- | --- | --- | --- | --- | --- | --- | --- | --- | --- | --- | --- | --- |
|  |  |  | **Older women?** | **Women at low risk of breast cancer recurrence?** | **Women at high risk of fracture?** | **Women with high risk of cardio-vascular disease?** | **Women at high risk of stroke?** | **Women at high risk of thrombo-embolism?** | **Women at high risk of endo-metrial cancer?** | **Women with multiple co-morbidities?** | **Sensitivity analysis for extrapolating beyond the follow-up time of studies** | **Sensitivity analysis for the discount rate** |
| 1 | Delea^1^ | Y | Y | N | N | N | N | N | N | N | Y | Y |
| 2 | Delea^2^ | Y | Y | N | N | N | N | N | N | N | Y | Y |
| 3 | Fonseca^3^ | N | N | N | N | N | N | N | N | N | N | N |
| 4 | Gamboa^4^ | Y | N | N | N | N | N | N | N | N | Y | Y |
| 5 | Gil^5^ | N | N | N | N | N | N | N | N | N | N | Y |
| 6 | Hillner^6^ | N | N | N | Y | N | N | N | N | Y | N | N |
| 7 | Hind^7^ | Y | Y | N | N | N | N | N | N | N | Y | Y |
| 8 | Karnon^8^ | Y | Y | Y | N | N | N | N | N | N | Y | Y |
| 9 | Lazarro^9^ | N | N | N | N | N | N | N | N | N | N/A | Y |
| 10 | Lee^10^ | Y | N | Y | N | N | N | N | N | N | N | Y |
| 11 | Locker^11^ | Y | N | N | N | N | N | N | N | N | Y | N |
| 12 | Lux^12^ | Y | N | N | N | N | N | N | N | N | Y | Y |
| 13 | Mansel^13^ | Y | N | N | N | N | N | N | N | N | Y | Y |
| 14 | Moeremans^14^ | N | N | N | N | N | N | N | N | N | N | N |
| 15 | Rocchi^15^ | N | N | N | N | N | N | N | N | N | Y | Y |
| 16 | Sasse^16^ | N | N | N | N | N | N | N | N | N | N | N |
| 17 | Skedgel^17^ | Y | Y | N | N | N | N | N | N | N | Y | Y |
| 18 | Skedgel^18^ | Y | Y | N | N | N | N | N | N | N | Y | Y |
