# Supplementary material for: A Systematic Review and Methodological Evaluation of Published Cost-Effectiveness Analyses of Aromatase Inhibitors versus Tamoxifen in Early Stage Breast Cancer
Source: PLoS One. 2013 May 6;8(5):e62614. doi: 10.1371/journal.pone.0062614 (PMC3646035; doi:10.1371/journal.pone.0062614)
Supplement: Appendix S1 — Terminology used to search literature databases. (DOC) [file pone.0062614.s005.doc]

**Appendix S1: Terminology used to search literature databases**

**Database: Ovid MEDLINE(R) In-Process & Other Non-Indexed Citations and Ovid MEDLINE(R) <1948 to Present>**

Search Strategy:

--------------------------------------------------------------------------------

1 exp Breast Neoplasms/ (179430)

2 exp Breast/ or exp Breast Diseases/ (205669)

3 exp Neoplasms/ (2185990)

4 2 and 3 (182778)

5 (cancer* adj3 breast*).tw. (141140)

6 (neoplas* adj3 breast*).tw. (1950)

7 (carcinoma* adj3 breast*).tw. (28809)

8 (adenocarcinoma* adj3 breast*).tw. (1830)

9 (tumour* adj3 breast*).tw. (3594)

10 (tumor* adj3 breast*).tw. (15560)

11 (malignan* adj3 breast*).tw. (5205)

12 5 or 6 or 7 or 8 or 9 or 10 or 11 (167309)

13 1 or 4 or 12 (218246)

14 exp Aromatase Inhibitors/ (4968)

15 aromatase inhibitor*.tw. (3852)

16 (Aminoglutethimide or orimeten or cytadren or fadrozole or afema or anastrozole or Arimidex or letrozole or femara or exemestane or aromasin or atamestane or finrozole or formestane or Lentaron or liarozole or minamestane or pyridoglutethimide or Vorozole or Rivizor).tw. (3668)

17 14 or 15 or 16 (7037)

18 13 and 17 (3716)

19 exp Tamoxifen/ (15669)

20 (Tamoxifen or Novaldex or tomaxithen or zitazonium or soltamox).tw. (15102)

21 19 or 20 (20119)

22 18 and 21 (2050)

23 Economics/ (25956)

24 exp "Costs and Cost Analysis"/ (154064)

25 "Value of Life"/ (5118)

26 "Quality of Life"/ (87932)

27 "cost of illness"/ (13573)

28 quality-adjusted life years/ (4795)

29 exp economics, hospital/ or exp economics, medical/ or economics, nursing/ or economics, pharmaceutical/ (35506)

30 exp "fees and charges"/ (25020)

31 exp models, economic/ (7651)

32 exp Budgets/ (10802)

33 ec.fs. (279502)

34 (costs or cost or costed or costly or costing*).tw. (249311)

35 (economic* or pharmacoeconomic* or price* or pricing).tw. (133145)

36 economic burden.tw. (2680)

37 cost effective*.tw. (56606)

38 quality of life.tw. (103000)

39 life quality.tw. (2588)

40 hql.tw. (71)

41 (Sf36 or sf thirtysix or sf thirty six or short form 36 or short form thirty six or short form thirtysix or short form 36).tw. (4732)

42 QOL.tw. (12579)

43 (euroqol or eq5d or eq 5d).tw. (2189)

44 QALY*.tw. (3334)

45 quality adjusted life year*.tw. (3830)

46 HYE*.tw. (544)

47 health* year* equivalen*.tw. (37)

48 health utilit*.tw. (739)

49 HUI.tw. (510)

50 quality of wellbeing*.tw. (7)

51 QWB.tw. (140)

52 quality of well being.tw. (272)

53 (QALD* or QALE* or Qtime*).tw. (80)

54 or/23-53 (708280)

55 22 and 54 (249)

56 limit 22 to ("costs (sensitivity)" or "costs (specificity)" or "costs (optimized)" or "economics (sensitivity)" or "economics (specificity)" or "economics (optimized)") (336)

57 55 or 56 (454)

58 remove duplicates from 57 (453)

59 limit 58 to yr="1996 -Current" (418)

**Database: EBM Reviews - Cochrane Database of Systematic Reviews <2005 to February 2011>, EBM Reviews - ACP Journal Club <1991 to February 2011>, EBM Reviews - Database of Abstracts of Reviews of Effects <1st Quarter 2011>, EBM Reviews - Cochrane Central Register of Controlled Trials <1st Quarter 2011>, EBM Reviews - Cochrane Methodology Register <1st Quarter 2011>, EBM Reviews - Health Technology Assessment <1st Quarter 2011>, EBM Reviews - NHS Economic Evaluation Database <1st Quarter 2011>**

Search Strategy:

--------------------------------------------------------------------------------

1 exp Breast Neoplasms/ (6797)

2 exp Breast/ or exp Breast Diseases/ (7329)

3 exp Neoplasms/ (39453)

4 2 and 3 (6823)

5 (cancer* adj3 breast*).tw. (12554)

6 (neoplas* adj3 breast*).tw. (1358)

7 (carcinoma* adj3 breast*).tw. (955)

8 (adenocarcinoma* adj3 breast*).tw. (57)

9 (tumour* adj3 breast*).tw. (199)

10 (tumor* adj3 breast*).tw. (395)

11 (malignan* adj3 breast*).tw. (190)

12 5 or 6 or 7 or 8 or 9 or 10 or 11 (13431)

13 1 or 4 or 12 (14069)

14 exp Aromatase Inhibitors/ (381)

15 aromatase inhibitor*.tw. (472)

16 (Aminoglutethimide or orimeten or cytadren or fadrozole or afema or anastrozole or Arimidex or letrozole or femara or exemestane or aromasin or atamestane or finrozole or formestane or Lentaron or liarozole or minamestane or pyridoglutethimide or Vorozole or Rivizor).tw. (1014)

17 14 or 15 or 16 (1157)

18 13 and 17 (849)

19 exp Tamoxifen/ (1616)

20 (Tamoxifen or Novaldex or tomaxithen or zitazonium or soltamox).tw. (2734)

21 19 or 20 (3217)

22 18 and 21 (490)

23 Economics/ (77)

24 exp "Costs and Cost Analysis"/ (30630)

25 "Value of Life"/ (298)

26 "Quality of Life"/ (11136)

27 "cost of illness"/ (3636)

28 quality-adjusted life years/ (2796)

29 exp economics, hospital/ or exp economics, medical/ or economics, nursing/ or economics, pharmaceutical/ (4159)

30 exp "fees and charges"/ (1114)

31 exp models, economic/ (2326)

32 exp Budgets/ (186)

33 ec.fs. (30556)

34 (costs or cost or costed or costly or costing*).tw. (52218)

35 (economic* or pharmacoeconomic* or price* or pricing).tw. (37151)

36 economic burden.tw. (490)

37 cost effective*.tw. (19655)

38 quality of life.tw. (26122)

39 life quality.tw. (537)

40 hql.tw. (18)

41 (Sf36 or sf thirtysix or sf thirty six or short form 36 or short form thirty six or short form thirtysix or short form 36).tw. (1286)

42 QOL.tw. (2868)

43 (euroqol or eq5d or eq 5d).tw. (1212)

44 QALY*.tw. (2811)

45 quality adjusted life year*.tw. (4398)

46 HYE*.tw. (18)

47 health* year* equivalen*.tw. (6)

48 health utilit*.tw. (392)

49 HUI.tw. (131)

50 quality of wellbeing*.tw. (1)

51 QWB.tw. (52)

52 quality of well being.tw. (242)

53 (QALD* or QALE* or Qtime*).tw. (49)

54 or/23-53 (75853)

55 22 and 54 (114)

56 limit 22 to ("costs (sensitivity)" or "costs (specificity)" or "costs (optimized)" or "economics (sensitivity)" or "economics (specificity)" or "economics (optimized)") [Limit not valid in CDSR,ACP Journal Club,DARE,CCTR,CLCMR; records were retained] (485)

57 55 or 56 (485)

58 remove duplicates from 57 (465)

59 limit 58 to yr="1996 -Current" [Limit not valid in DARE; records were retained] (412)

**Database: EMBASE <1980 to 2011 Week 09>**

Search Strategy:

--------------------------------------------------------------------------------

1 exp breast tumor/ (255720)

2 exp BREAST/ or exp BREAST DISEASE/ (301071)

3 exp neoplasm/ (2550949)

4 2 and 3 (264347)

5 (cancer* adj3 breast*).tw. (167817)

6 (neoplas* adj3 breast*).tw. (2151)

7 (carcinoma* adj3 breast*).tw. (31854)

8 (adenocarcinoma* adj3 breast*).tw. (2010)

9 (tumour* adj3 breast*).tw. (4276)

10 (tumor* adj3 breast*).tw. (17784)

11 (malignan* adj3 breast*).tw. (5969)

12 5 or 6 or 7 or 8 or 9 or 10 or 11 (196504)

13 1 or 4 or 12 (287003)

14 exp aromatase inhibitor/ (14741)

15 aromatase inhibitor*.tw. (4920)

16 (Aminoglutethimide or orimeten or cytadren or fadrozole or afema or anastrozole or Arimidex or letrozole or femara or exemestane or aromasin or atamestane or finrozole or formestane or Lentaron or liarozole or minamestane or pyridoglutethimide or Vorozole or Rivizor).tw. (5785)

17 14 or 15 or 16 (15455)

18 13 and 17 (9541)

19 exp TAMOXIFEN CITRATE/ or exp TAMOXIFEN/ or exp TAMOXIFEN AZIRIDINE/ or exp TAMOXIFEN DERIVATIVE/ (37707)

20 (Tamoxifen or Novaldex or tomaxithen or zitazonium or soltamox).tw. (18393)

21 19 or 20 (39650)

22 18 and 21 (6582)

23 ECONOMICS/ (184469)

24 exp health economics/ (489479)

25 exp "health care cost"/ (157921)

26 medical fee/ (9529)

27 exp "quality of life"/ (170804)

28 budget/ (15239)

29 (costs or cost or costed or costly or costing*).tw. (296744)

30 (economic* or pharmacoeconomic* or price* or pricing).tw. (155382)

31 economic burden.tw. (3379)

32 quality of life.tw. (132445)

33 life quality.tw. (3978)

34 hql.tw. (84)

35 (Sf36 or sf thirtysix or sf thirty six or short form 36 or short form thirty six or short form thirtysix or short form 36).tw. (5541)

36 QOL.tw. (17539)

37 (euroqol or eq5d or eq 5d).tw. (2958)

38 QALY*.tw. (4260)

39 quality adjusted life year*.tw. (4542)

40 HYE*.tw. (769)

41 health* year* equivalen*.tw. (41)

42 health utilit*.tw. (865)

43 HUI.tw. (585)

44 quality of wellbeing*.tw. (11)

45 QWB.tw. (153)

46 quality of well being.tw. (299)

47 quality of well being.tw. (299)

48 (QALD* or QALE* or Qtime*).tw. (99)

49 or/23-48 (997103)

50 22 and 49 (1106)

51 limit 50 to yr="1996 -Current" (1081)

52 limit 22 to ("economics (1 term high sensitivity)" or "economics (1 term high specificity)" or "economics (1 term min difference)" or "economics (2 or more terms high sensitivity)" or "economics (2 or more terms high specificity)" or "economics (2 or more terms min difference)") (934)

53 limit 52 to yr="1996 -Current" (888)

54 51 or 53 (1555)

55 remove duplicates from 54 (1496)

Database: PsycINFO <1987 to March Week 1 2011>

Search Strategy:

--------------------------------------------------------------------------------

1 exp Breast Neoplasms/ (4801)

2 exp Breast/ or exp Breast Diseases/ (469)

3 exp Neoplasms/ (22753)

4 2 and 3 (139)

5 (cancer* adj3 breast*).tw. (6140)

6 (neoplas* adj3 breast*).tw. (28)

7 (carcinoma* adj3 breast*).tw. (73)

8 (adenocarcinoma* adj3 breast*).tw. (5)

9 (tumour* adj3 breast*).tw. (7)

10 (tumor* adj3 breast*).tw. (61)

11 (malignan* adj3 breast*).tw. (43)

12 5 or 6 or 7 or 8 or 9 or 10 or 11 (6209)

13 1 or 4 or 12 (6336)

14 exp Aromatase Inhibitors/ (0)

15 aromatase inhibitor*.tw. (117)

16 (Aminoglutethimide or orimeten or cytadren or fadrozole or afema or anastrozole or Arimidex or letrozole or femara or exemestane or aromasin or atamestane or finrozole or formestane or Lentaron or liarozole or minamestane or pyridoglutethimide or Vorozole or Rivizor).tw. (99)

17 14 or 15 or 16 (162)

18 13 and 17 (20)

19 exp Tamoxifen/ (0)

20 (Tamoxifen or Novaldex or tomaxithen or zitazonium or soltamox).tw. (242)

21 19 or 20 (242)

22 18 and 21 (16)

23 Economics/ (9877)

24 exp "Costs and Cost Analysis"/ (12641)

25 "Value of Life"/ (0)

26 "Quality of Life"/ (18725)

27 "cost of illness"/ (0)

28 quality-adjusted life years/ (0)

29 exp economics, hospital/ or exp economics, medical/ or economics, nursing/ or economics, pharmaceutical/ (0)

30 exp "fees and charges"/ (0)

31 exp models, economic/ (0)

32 exp Budgets/ (447)

33 ec.fs. (0)

34 (costs or cost or costed or costly or costing*).tw. (42380)

35 (economic* or pharmacoeconomic* or price* or pricing).tw. (51557)

36 economic burden.tw. (368)

37 cost effective*.tw. (6711)

38 quality of life.tw. (27915)

39 life quality.tw. (789)

40 hql.tw. (16)

41 (Sf36 or sf thirtysix or sf thirty six or short form 36 or short form thirty six or short form thirtysix or short form 36).tw. (983)

42 QOL.tw. (3927)

43 (euroqol or eq5d or eq 5d).tw. (563)

44 QALY*.tw. (417)

45 quality adjusted life year*.tw. (462)

46 HYE*.tw. (246)

47 health* year* equivalen*.tw. (4)

48 health utilit*.tw. (220)

49 HUI.tw. (244)

50 quality of wellbeing*.tw. (7)

51 QWB.tw. (71)

52 quality of well being.tw. (148)

53 (QALD* or QALE* or Qtime*).tw. (5)

54 or/23-53 (117248)

55 22 and 54 (10)

56 limit 22 to ("costs (sensitivity)" or "costs (specificity)" or "costs (optimized)" or "economics (sensitivity)" or "economics (specificity)" or "economics (optimized)") [Limit not valid in PsycINFO; records were retained] (16)

57 55 or 56 (16)

58 remove duplicates from 57 (16)

59 limit 58 to yr="1996 -Current" (16)
