# Supplementary material for: A Systematic Review and Methodological Evaluation of Published Cost-Effectiveness Analyses of Aromatase Inhibitors versus Tamoxifen in Early Stage Breast Cancer
Source: PLoS One. 2013 May 6;8(5):e62614. doi: 10.1371/journal.pone.0062614 (PMC3646035; doi:10.1371/journal.pone.0062614)
Supplement: Appendix S2 — References to the online supplemental documents. (DOC) [file pone.0062614.s006.doc]

**References**

1. Delea TE, Karnon J, Sofrygin O, Thomas SK, Papo NL, Barghout V, et al. Cost-effectiveness of letrozole versus tamoxifen as initial adjuvant therapy in hormone receptor-positive postmenopausal women with early-stage breast cancer. Clinical Breast Cancer 2007;7(8):608-18.

2. Delea TE, El-Ouagari K, Karnon J, Sofrygin O, Delea TE, El-Ouagari K, et al. Cost-effectiveness of letrozole versus tamoxifen as initial adjuvant therapy in postmenopausal women with hormone-receptor positive early breast cancer from a Canadian perspective. Breast Cancer Research & Treatment 2008;108(3):375-87.

3. Fonseca M, Araujo GT, Saad ED, Fonseca M, Araujo GTB, Saad ED. Cost-effectiveness of anastrozole, in comparison with tamoxifen, in the adjuvant treatment of early breast cancer in Brazil. Revista Da Associacao Medica Brasileira 2009;55(4):410-5.

4. Gamboa O, Diaz S, Chicaiza L, Garcia M. [Cost-benefit analysis of anastrazol and tamoxifen in adjuvant treatment of hormone receptor-positive, post-menopausal breast cancer]. [Spanish]. Biomedica 2010;30(1):46-55.

5. Gil JM, Rubio-Terres C, Del Castillo A, Gonzalez P, Canorea F, Gil JM, et al. Pharmacoeconomic analysis of adjuvant therapy with exemestane, anastrozole, letrozole or tamoxifen in postmenopausal women with operable and estrogen receptor-positive breast cancer. Clinical & Translational Oncology: Official Publication of the Federation of Spanish Oncology Societes & of the National Cancer Institute of Mexico 2006;8(5):339-48.

6. Hillner BE. Benefit and projected cost-effectiveness of anastrozole versus tamoxifen as initial adjuvant therapy for patients with early-stage estrogen receptor-positive breast cancer. Cancer 2004;101(6):1311-22.

7. Hind D, Ward S, De Nigris E, Simpson E, Carroll C, Wyld L, et al. Hormonal therapies for early breast cancer: systematic review and economic evaluation. Health Technology Assessment (Winchester, England);11(26):iii-iv.

8. Karnon J, Delea T, Barghout V, Karnon J, Delea T, Barghout V. Cost utility analysis of early adjuvant letrozole or anastrozole versus tamoxifen in postmenopausal women with early invasive breast cancer: the UK perspective. European Journal of Health Economics 2008;9(2):171-83.

9. Lazzaro C. Cost-utility analysis of anastrozole versus tamoxifen for adjuvant treatment in postmenopausal women with early breast cancer. [Italian]. PharmacoEconomics - Italian Research Articles 2007;9(1):31-43.

10. Lee HJ, Lee TJ, Yang BM, Min J. Cost-effectiveness analysis of adjuvant hormonal treatments for women with postmenopausal hormone-receptor positive early breast cancer in the Korean context. Journal of Breast Cancer;13(3):286-98.

11. Locker GY, Mansel R, Cella D, Dobrez D, Sorensen S, Gandhi SK, et al. Cost-effectiveness analysis of anastrozole versus tamoxifen as primary adjuvant therapy for postmenopausal women with early breast cancer: a US healthcare system perspective. The 5-year completed treatment analysis of the ATAC ('Arimidex', Tamoxifen Alone or in Combination) trial. Breast Cancer Research & Treatment 2007;106(2):229-38.

12. Lux MP, Wockel A, Benedict A, Buchholz S, Kreif N, Harbeck N, et al. Cost-effectiveness analysis of anastrozole versus tamoxifen in adjuvant therapy for early-stage breast cancer - a health-economic analysis based on the 100-month analysis of the ATAC trial and the German health system. Onkologie 2010;33(4):155-66.

13. Mansel R, Locker G, Fallowfield L, Benedict A, Jones D, Mansel R, et al. Cost-effectiveness analysis of anastrozole vs tamoxifen in adjuvant therapy for early stage breast cancer in the United Kingdom: the 5-year completed treatment analysis of the ATAC ('Arimidex', Tamoxifen alone or in combination) trial. British Journal of Cancer 2007;97(2):152-61.

14. Moeremans K, Annemans L. Cost-effectiveness of anastrozole compared to tamoxifen in hormone receptor-positive early breast cancer. Analysis based on the ATAC trial. International Journal of Gynecological Cancer 2006;16 Suppl 2:576-8.

15. Rocchi A, Verma S. Anastrozole is cost-effective vs tamoxifen as initial adjuvant therapy in early breast cancer: Canadian perspectives on the ATAC completed-treatment analysis. Supportive Care in Cancer 2006;14(9):917-27.

16. Sasse AD, Sasse EC. [Cost-effectiveness analysis of adjuvant anastrozol in post-menopausal women with breast cancer]. [Portuguese]. Revista Da Associacao Medica Brasileira 2009;55(5):535-40.

17. Skedgel C, Rayson D, Dewar R, Younis T, Skedgel C, Rayson D, et al. Cost-utility of adjuvant hormone therapies for breast cancer in post-menopausal women: sequential tamoxifen-exemestane and upfront anastrozole. Breast Cancer Research & Treatment 2007;101(3):325-33.

18. Skedgel C, Rayson D, Dewar R, Younis T, Skedgel C, Rayson D, et al. Cost-utility of adjuvant hormone therapies with aromatase inhibitors in post-menopausal women with breast cancer: upfront anastrozole, sequential tamoxifen-exemestane and extended tamoxifen-letrozole. Breast 2007;16(3):252-61.

19. Neumann PJ, Stone PW, Chapman RH, Sandberg EA, Bell CM. The quality of reporting in published cost-utility analyses, 1976-1997.[see comment]. Annals of Internal Medicine. 2000; **132**(12): 964-72.
